# Health-Related Quality of Life in Juvenile Idiopathic Arthritis: A Systematic Review of Phase III Clinical Trials

**DOI:** 10.3390/jcm14010254

**Published:** 2025-01-03

**Authors:** Federica Romano, Federica Di Scipio, Giacomo Baima, Francesco Franco, Mario Aimetti, Giovanni Nicolao Berta

**Affiliations:** 1 Department of Surgical Sciences, C.I.R. Dental School, University of Turin, Via Nizza 230, 10126 Turin, Italy; federica.discipio@unito.it (F.D.S.); giacomo.baima@unito.it (G.B.); mario.aimetti@unito.it (M.A.); 2 Department of Clinical and Biological Sciences, Section of Translational Pharmacology, University of Turin, Regione Gonzole 10, 10043 Orbassano, Italy; francesco.franco@unito.it

**Keywords:** juvenile idiopathic arthritis, health-related quality of life, patient-reported outcomes, phase III trials, endpoints

## Abstract

**Background/objectives:** Juvenile idiopathic arthritis (JIA) is the most common rheumatic disease in childhood, leading to severe disability and negatively affecting patients’ health-related quality of life (HRQoL). The aim of this systematic review was to evaluate the adoption, reporting and assessment methodology of HRQoL in phase III clinical trials involving children with JIA. **Methods:** An electronic and manual search was conducted to identify primary and secondary publications of pharmacological trials conducted between 2012 and 2023. Data were extracted and recorded in duplicate. **Results:** A total of 222 studies were screened and 24 articles (22 primary and 2 secondary publications) were included in the review. HRQoL was not listed among the endpoints in 10 trials (45.5%), while it was a secondary endpoint in 12 trials (54.5%). The proportion of trials that did not consider HRQoL was equally relevant in both for-profit and no-profit settings (44.4% versus 50.0%), but it was higher in studies on systemic JIA compared to other JIA subtypes (62.5%), and on IL inhibitor treatment (72.7%) with respect to other disease-modifying antirheumatic drugs. Information on HRQoL was usually collected from parents/caregivers, and only three studies were categorized as “probably robust” with regard to HRQoL assessment. **Conclusions:** Systematic incorporation of HRQoL measures represents an urgent need in pediatric rheumatology, aiding clinicians in their decision-making in relation to treatment effectiveness and considering the children’s perspective.

## 1. Introduction

Juvenile idiopathic arthritis (JIA) is the most common chronic rheumatic disease in childhood, with estimated prevalence and annual incidence rates ranging from 3.8 to 400 and from 0.8 to 23 per 100,000 children, respectively [1]. This umbrella term includes a clinically heterogeneous group of disorders of unknown etiology, but with joint inflammation, which begins prior to the age of 16 years and persists for longer than 6 weeks, as their main hallmark [2,3]. Systemic JIA (sJIA), oligoarthritis (oligoJIA), rheumatoid factor (RF)-negative polyarthritis (poliJIA RF^−^), RF-positive polyarthritis (poliJIA RF^+^), juvenile psoriatic arthritis (PsJIA), enteritis-related arthritis (ERA), and undifferentiated arthritis are the seven subtypes recognized under the current classification by the International League of Associations for Rheumatology (ILAR) [2].

The persisting inflammation is responsible for morning stiffness, swollen joints, disturbance in functions, and pain, limiting the patient’s daily activities and productivity compared to their healthy peers [4]. The involvement of the temporomandibular joint, occurring in approximately 40% of children, limits jaw movements and decreases mouth-opening capacity, affecting mastication and leading to dento-facial growth disturbance in the most severe cases [5]. Besides the joints, extra-articular structures, including eyes, skin, and internal organs, can be involved [3,6]. Salivary hypofunction has also been reported, predisposing patients to tooth decay [7].

As a result of chronic pain and physical/functional disability, JIA has a negative impact on the health-related quality of life (HRQoL) of the affected children. HRQoL is a more specific term than QoL, as it focuses on the correlation between the health and functioning of an individual [8]. It represents a crucial outcome of chronic health conditions, and its improvement should be one of the main goals of patient care [9]. Indeed, treatment strategies for JIA should aim to control/relieve pain, improve physical function, manage systemic complications, and improve HRQoL, supporting children’s psychosocial development [10].

Pharmacologic therapy has traditionally involved the administration of non-steroidal anti-inflammatory drugs (NSAIDs) and intra-articular glucocorticoids; however, recent advances in JIA management include the use of conventional synthetic and biologic disease-modifying antirheumatic drugs (DMARDs) according to the JIA subtype [11]. Methotrexate is the conventional synthetic (cs)DMARD that is most commonly used and it remains the cornerstone of JIA treatment, providing clinical benefits with an acceptable profile of toxic effects [12]. Advanced biologics (bDMARDSs), which target specific cytokine or cell–cell interactions, are recommended as the second line of treatment in patients experiencing continuous disease progression [13]. Indeed, tumor necrosis factor (TNF)-α inhibitors (TNFi) and interleukin (IL)-1β signaling blockade agents are important treatment options, considering the pivotal role of these pro-inflammatory mediators in JIA pathogenesis [14,15]. Janus-associated tyrosine kinase (JAK) inhibitors represent a new, promising class of drugs, which block the production of selected cytokines by interrupting the transduction of extracellular pro-inflammatory signals into the cell nucleus.

Although bDMARDs have changed the outlook of JIA patients, as they may help to control disease activity, there is still a need for research supporting the development of new pharmacological agents that are able to increase the percentage of disease remission [16]. Complete suppression of joint inflammation and low disease activity are difficult to achieve, and about half of patients continue to require active treatment in their adult life [17]. Thus, phase III clinical trials are warranted, as they will provide scientifically sound and statistically robust evidence about the safety and effectiveness of new treatment approaches prior to marketing authorization [18]. In addition to traditional clinical outcomes, influential organizations like the National Institute for Health and Care Excellence recommend the use of HRQoL measures in clinical trials to assess the magnitude of treatment response [19]. The use parent-/child-reported outcomes (PCROs) to assess the HRQoL before, during, and after a pharmacological treatment can help to measure the impact of the drugs and to demonstrate a tangible clinical benefit for patients [20]. Nonetheless, HRQoL assessment is still not frequently incorporated among studies’ endpoints.

Thus, the primary aim of this systematic review was to assess whether HRQoL was included among the endpoints of phase III clinical trials testing the efficacy of pharmacological interventions for JIA management. The secondary aim was to address the potential methodological issues associated with HRQoL assessment.

## 2. Materials and Methods

### 2.1. Search Strategy for the Identification of the Studies

The present review was reported according to the Preferred Reporting Items for Systematic Review and Meta-Analyses (PRISMA) statement [21] and we aimed to answer the following focused question: “In pediatric patients, was HRQoL considered as primary or secondary endpoint in phase III clinical trials testing the efficacy of experimental pharmacological treatment for JIA?”.

A systematic literature search was conducted via the online databases Medline (via PubMed), Scopus, and Web of Science using a strategy based on a combination of Medical Subject Headings (MeSH) and free text words, without any limit on the publication status (Supplementary Table S1). The search was restricted to publications in English from January 2012 to December 2023. This 12-year time frame was chosen to capture the most recent and relevant advancements in the field, aligning with significant developments in JIA. Additionally, five major journals in the rheumatology field (*The Lancet Rheumatology*, *Annals of the Rheumatic Diseases*, *Arthritis and Rheumatology*, *Rheumatology*, and *Arthritis Research and Therapy*) were hand-searched.

After duplicates were removed using reference management software (EndNote^TM^, Thomson Reuters, Philadelphia, PA, USA), the title and abstract were screened by two independent reviewers, followed by full-text assessment of potentially relevant records. Disagreements on the publications’ eligibility were resolved by discussion and, when necessary, by consulting a third reviewer. Reference lists of the included publications were also reviewed to identify any additional article of possible relevance.

### 2.2. Criteria for Considering Studies for This Review

Criteria for study selection were based on the PICOS method.

#### 2.2.1. Types of Participants (P)

Participants were pediatric patients of both sexes, aged ≤ 18 years, who had been diagnosed with JIA disease according to the ILAR criteria [2]. All JIA subgroups were eligible.

#### 2.2.2. Types of Interventions (I)

Oral, parenteral or injective drug alone or in combination with standard treatment was considered without any restrictions regarding the dosage or duration of the intervention.

#### 2.2.3. Types of Comparisons (C)

Any standard treatment, placebo, or lack thereof was taken into consideration.

#### 2.2.4. Types of Outcomes (O)

HRQoL measured with any questionnaire or visual analogue (VAS) scale was considered (either as a primary, secondary, or exploratory endpoint). Only the patient’s self-reported measures were taken into account due to the large variability in HRQoL results between physician-assessed and patient self-assessed outcomes [22].

#### 2.2.5. Types of Studies (S)

Only phase III clinical trials were selected. No restriction in the number of patients enrolled in the trial was applied. The search was restricted to randomized controlled clinical trials (RCTs), controlled clinical trials (CCTs) or single-arm longitudinal trials. Observational studies, abstracts, registries, or non-peer-reviewed publications, as well as trials testing non-pharmacological interventions, were excluded from the analysis.

### 2.3. Data Extraction

Two reviewers independently collected data from each selected article. The extracted data were double-checked and any discrepancy was resolved by consensus. The following data were recorded on a standardized spreadsheet:

- Publication: journal, first author, and year of publication;
- Population: number of patients, age at baseline, and type of JIA;
- Trial design and conduction: single versus multi-center study, study location (single country versus two or more countries), sponsorship (no-profit if sponsored by an academic institution or a cooperative group versus for-profit if industry-funded), type of design (RCT, single-arm study, long-term extension of phase III study), allocation concealment (open-label versus blinded), and details of pharmacological treatment in both experimental and control arms;
- Study endpoints: HRQoL included among primary/secondary/explorative endpoints as derived from the Section 2 or Section 3 of the publication and/or from the study protocol, the type of instrument adopted, and overall differences in HRQoL between the experimental and control group.

Each included study was classified as low (<10), intermediate (10–20) and high according (>20) to the journal’s impact factor (IF) retrieved from the *Journal of Citation Reports* in the year of publication. It was also categorized as “positive” or “negative” according to the result of the primary endpoint. In addition, drug treatments were divided into four main groups: Janus Kinase inhibitors, TNF inhibitors, IL inhibitors, and B/T cell functioning inhibitors. Finally, the number of rows allocated to HRQoL description in the Section 3 was computed and the presence of HRQoL restricted to Tables or Figures in the main text or in the Supplementary Materials was also recorded.

### 2.4. Study Quality Assessment and Impact of HRQoL Results on Clinical Decision-Making

Due to the lack of a specific instrument for JIA, the methodological quality of the included articles was assessed by one reviewer according to the Minimum Standard Checklist for evaluating HRQoL outcomes in clinical trials (MSC) [23]. For the purpose of quality control, a second reviewer independently classified the articles. In cases of disagreement, articles were discussed and a final decision was reached. The MSC checklist is organized into 11 items divided into four categories: conceptual (a priori hypothesis, rationale for the instrument), measurement (psychometric properties, cultural validity, and adequacy of the domains), methodology (instrument administration, baseline compliance, time of assessment and missing data) and interpretation (clinical significance and presentation of the results). Every item has a dichotomous answer, with “yes” score representing one point. The scores are added together, ranging from 0 to 11, and based on the overall checklist score, each study is rated as “probably robust” (indicated by a score between 8 and 11), “limited” (indicated by a score between 5 and 7) or “very limited” (indicated by a score between 0 and 4) [23].

Finally, the impact of HRQoL results on clinical decision-making was scored based on the criteria proposed by Kvam et al. [24]. Studies were categorized according to (i) whether the authors claimed that HRQoL outcome should influence clinical decision-making and (ii) whether HRQoL measurement was methodologically evaluated as “probably robust” and likely to provide useful data to further facilitate clinical decision-making.

## 3. Results

### 3.1. Study Selection

**Figure 1:**
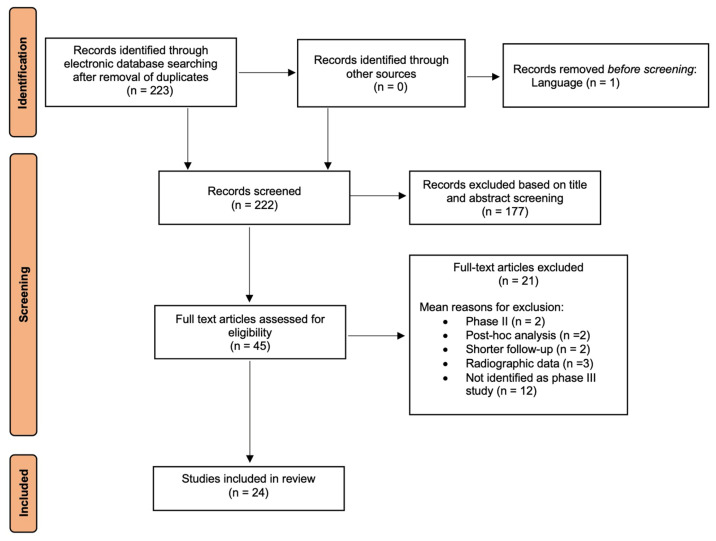
PRISMA flow diagram for screening and identification of publications.

The electronic and manual search provided a total of 223 studies after the removal of duplicates; only 1 article was discarded because it was not written in the English language (Figure 1).

A total of 222 studies were screened: of these, 177 were excluded after first-stage reading of titles and abstracts and 21 articles were removed after full-text reading. Finally, 24 articles (22 primary and 2 secondary publications) were included in the qualitative analysis. The inter-examiner reliability was excellent in both the screening and inclusion process (κ score = 0.85, 95% CI: 0.81–0.89) and the full-text appraisal (κ score = 0.91; 95% CI, 0.88–0.94).

### 3.2. Study Characteristics

The main characteristics of the primary publications of phase III clinical trials included in this review are summarized in Table 1. Most of the studies (45.4%) were published between 2012 and 2015 and in high-impact-factor journals (40.9%). The majority of the trials (59.09%) were conducted by members of the Pediatric Rheumatology International Trials Organization (PRINTO) and the Pediatric Rheumatology Collaborative Study Group (PRCSG) in patients with sJIA or a polyarticular course of JIA (including poliJIA, extended oligoJIA, ERA and PsJIA). IL inhibitor therapy (mainly antiIL-1 and antiIL-6 agents) was the most commonly studied treatment (50.0%), followed by anti-TNF therapy (27.3%). All studies but one reported positive results and approximately 80% of them were sponsored by drug companies.

The details of each primary publication of the eligible phase III trials are reported in Table 2.

### 3.3. Inclusion of HRQoL Among Study Endpoints

The inclusion of HRQoL among primary/secondary endpoints according to the study characteristics of the primary publications is summarized in Table 3. No study reported HRQoL as the primary endpoint, but almost all of them considered the JIA/ACR 30 as a primary outcome (see Table 2). This composite outcome scores the overall disease activity/response rate based on six JIA core set variables; three of them are requested to improve by at least 30% in order for the treatment to be considered successful, with no more than one of the remaining variables worsening by 30% or more. Indeed, the parent/patient’s global assessment of overall well-being and functional ability were recorded as part of the JIA/ACR core set variables (in addition to the physician’s evaluation of disease activity, number of joints with active arthritis, number of joints with limited motion, and erythrocyte sedimentation rate).

Overall, HRQoL was a secondary endpoint in 12 trials (54.5%), while in the remaining 10 (45.5%) it was not listed at all among the study endpoints. HRQoL was not included as an endpoint in 37.5%, 40.0%, and 55.6% of the papers published in low-, intermediate-, and high-JIF journals, respectively. The proportion of trials that did not include HRQoL data was relevant in both for-profit and no-profit settings (44.4% versus 50.0%) and it was similar over time (33.3% in the years 2020–2023 compared to 40% in the years 2012–2015). It was higher in studies on sJIA compared to other JIA subtypes (62.5%) and on IL inhibitor treatment (72.7%) compared to other DMARDs.

The majority of the studies reported the main HRQoL results in a few lines throughout the Section 3 of the manuscript (median space: 11.5 rows) or summarized them only in Tables or Figures. The space allocated to HRQoL details in older publications was shorter than that dedicated in the most recent ones. One study did not report any data in the paper, although HRQoL was listed among its endpoints [40].

Finally, two secondary publications were identified on IL-6 inhibitor therapy in sJIA and polycourse JIA, which reported HRQoL data as primary endpoint after a mean publication time of 102 months [36,48].

### 3.4. Methodology for HRQoL Assessment and Reporting

Data on HRQoL methodology, including the instruments adopted, type of analysis, and presentation of the results, are summarized in Table 4, referring to primary and secondary publications of trials assessing HRQoL. Two studies included secondary analysis of previously published data [36,48]. More detailed information is available in Supplementary Table S2. The most common JIA-specific tools adopted were the Patient/Parent Global Assessment (PtGA; 23.1%) and the Child Health Assessment Questionnaire (CHAQ; 61.5%), the first was applied for HRQoL evaluation, while the second one was used to measure functional outcomes, mainly for the disability index component. Moreover, individual perception of overall pain and/or nocturnal back pain was recorded on a 0–100 VAS scale in more than half of the studies (53.8%). Information on HRQoL was obtained from the parents/caregivers, irrespective of the age of the enrolled patients. Only the study by Brunner et al. specified that the questionnaires were completed by adolescent patients themselves or by parents for younger children [48].

The methods of analysis more commonly used were mean/median values or changes from baseline in overall or domain-specific tool scores. In addition, only four studies provided an explicit statement about the statistical approaches adopted to deal with missing data and only five provided any explicit definition of minimal clinically important difference (MCID) in HRQoL scores.

### 3.5. Quality of HRQoL Assessment and Impact of HRQoL Results on Clinical Decision-Making

The checklist score for each HRQoL assessment according to the Minimum Standard Checklist for evaluating HRQoL outcomes in clinical trials is reported in Table 5 (additional information is available in Supplementary Table S3). No study listed HRQoL among the study primary endpoints and only two studies reported an a priori hypothesis.

Regarding HRQoL measurement, the cultural validity of the questionnaires was verified in most of the included studies, while no study provided information about the administration of HRQoL measurement (i.e., by telephone, by computer touch screen, or administered by health care professionals). In contrast, the timing of the HRQoL assessment and information about baseline compliance were reported in the majority of the studies. With regard to the interpretation, the studies presented HRQoL results in general terms, and only a minority of them also discussed the clinical relevance of the outcomes.

Based on their global score, four studies could be considered as very limited in terms of their methodological design, according to previously defined criteria (defined by a score between 0 and 4). Six trials were rated as limited (defined by a score between 5 and 7), while three were judged as being probably robust (defined by a score between 8 and 11) and were likely to provide useful data to further facilitate clinical decision-making.

## 4. Discussion

The main objective of this systematic review was to assess the prevalence of HRQoL among the reported outcomes of clinical trials conducted in pediatric patients with JIA and to evaluate their methodological quality. By focusing on the phase III clinical setting, we aimed to provide the most up-to-date evidence regarding the importance assigned to the patient’s HRQoL in efficacy trials for JIA, and the possible added value of this research in supporting clinical decision-making. Because JIA is a chronic disease associated with severe symptoms, there is no cure, but remission should be a major aim of treatment, along with maintaining quality of life.

Using the stated selection and eligibility criteria, we found, overall, 22 phase III trials conducted over the last 12 years, mainly with a randomized withdrawal design, and only 12 of these (54.5%) involved HRQoL assessment [49]. Although HRQoL was not included as an endpoint in a relevant proportion of these studies, almost all trials with a HRQoL component reported the corresponding results in the primary publication. This highlights a pressing need for standardization in the adoption and reporting of HRQoL outcomes to improve consistency and usability across trials.

As expected, changes in disease activity and disease state were the primary response outcomes used to measure treatment efficacy in all the selected studies [50]. Considering the complexity of JIA, disease assessment was based on composite measures, which incorporate multiple items to capture different clinical manifestations. Indeed, JIA/ACR Pedi 30, which takes into account six criteria approved by the American College of Rheumatology, was considered as primary endpoint in the majority of the studies [50]. This instrument has been recognized by both the US Food and Drug Administration and the European Medicines Agency as an efficacy response measure for registration trials [51]. Nonetheless, its clinical utility may be limited due to its dichotomous and relative nature, being defined as 30% improvement in disease activity with respect to baseline values, and the lack of information regarding the patient’s disease state and perception [52].

In the last few years, the scientific community has expressed a growing interest in the impact of drug therapy on patients’ well-being and everyday life in JIA, and PCROs have gained increasing recognition as important tools for monitoring the disease course [53]. Previous studies on other chronic conditions such as asthma, diabetes mellitus, and autoimmune diseases have shown improved treatment adherence when patient perception was integrated into the decision-making process [54,55]. This type of shared decision-making approach was also endorsed by the international task force for the treatment of JIA in 2018 [56]. Nevertheless, despite an evident improvement in the most recent years (33.3% between 2020 and 2023 compared to 66.7% between 2016 and 2019), the proportion of trials including HRQoL remained suboptimal in the time frame considered in this systematic review. It should be taken into consideration that PRO-specific guidelines for clinical trial protocols were included into the SPIRIT (Standard Protocol Items: Recommendations for Interventional Trials) statement in 2018 [57].

The absence of HRQoL among the study endpoints was commonly detected both in industry and academia/independent cooperative group sponsored trials, and was even found to be slightly higher among the latter category (44.4% versus 50.0%). This finding is surprising if we consider that academic researchers should be more prone to collecting HRQoL data than drug companies, as these data are useful for informing the best clinical practice and play a role in the shared decision-making process. Similar figures were reported in previous phase III studies on solid cancer treatment [58,59,60].

Interestingly, trials published in highly ranked journals included HRQoL among secondary endpoints less frequently than studies published in journals with intermediate or low IFs. It is difficult to interpret this finding because higher-IF journals are expected to publish well-designed and high-quality clinical trials, which should usually include HRQoL among the outcome measures, due to their more stringent publication requirements. Notably, in this analysis, most of them were general medical journals, while rheumatic disease journals were prevalent in low- and intermediate-IF categories.

Moreover, the proportion of studies without HRQoL assessment was higher in sJIA settings compared to other JIA subtypes (62.5%) and in IL inhibitor therapeutic modality (72.7%) with respect to other more recently introduced DMARDs. In line with a recent publication [61], more than one-third of the studies included in this systematic review specifically addressed the sJIA subset, which represents the most serious disease subtype [62]. Due to the presence of both systemic and joint inflammation, these patients are at risk of progressive joint damage and growth impairment, but also of life-threatening complications, which can occur at any time during the disease course. Despite the recent considerable therapeutic advances, there remain subsets of sJIA with refractory disease and severe disease-associated complications [63].

Finally, we found that HRQoL reporting occupied a limited space in the Section 3 of the primary publications, with a median length of 11.5 rows, but with a trend of improvement over the 12-year time frame. In agreement with this result, a previous systematic review reported that the median space allocated to PRO details in phase III cancer RCTs was 12, but in contrast with the present results, it did not envisage any substantial improvement over time [58]. In our review, only two HRQoL reports were published in a separate publication from the original trials, which assessed the efficacy of IL-6 inhibitor therapy in sJIA and polycourse JIA [36,48]. Reale et al. emphasized that this strategy may decrease the interest in HRQoL results, with the possibility that they will not be published, that they will be published in low-impact journals, or that there will be significant delays in their publication [59]. In fact, despite the advantage of providing a more detailed description of the HRQoL results (63 and 140 rows, respectively), such secondary publications made data available after a mean delay of 102 months in a low-IF journal.

The second objective of this review was to evaluate the methodological quality of HRQoL assessment and the impact of HRQoL findings on treatment recommendations. Assessing HRQoL results from trials is difficult due to the use of different generic or disease-specific tools, timelines, analytical approaches, and metrics for interpretation (MCID). In a critical methodological assessment, only three studies were categorized as “probably robust” in terms of HRQoL and were deemed potentially useful for influencing clinical decision-making. Two of these studies were published in 2021 and 2023, suggesting an improvement in the quality of HRQoL reporting over the last few years.

None of the trials with a secondary HRQoL outcome explicitly stated an a priori hypothesis related to HRQoL. This leads us to question whether these studies were adequately powered to demonstrate changes as measured by the chosen PCRO instrument. Multiple significance testing, if not pre-specified, may lead to false-positive results, increasing the chance of type I error [64]. At the same time, only five papers reported the MICD of all the HRQoL scores analyzed, which is fundamental if the study results are to be deemed clinically significant from a patient’s perspective. Thus, for a sufficiently large sample size, it is possible that a difference could be so small that it would be not clinically relevant, even if it was statistically significant [65]. This makes interpreting the clinical meaningfulness of results challenging.

Compliance and missing data are also important issues. Children with JIA are often severely ill and it is possible that they are not compliant with PCRO assessment. In such situations, the missing data are not random and may no longer be representative. Notably, only four studies provided an explicit statement about the statistical approaches adopted to deal with missing data.

We found significant heterogeneity in the methods used for the analysis and presentation of HRQoL results. Mean/median scores or mean changes from baseline were commonly used to summarize data. However, this descriptive method does not take into account between-subject variability, hence, it cannot capture the heterogeneity in the individual HRQoL experience [66]. Analysis of responders in each HRQoL domain would provide more relevant information, but in the present analysis, this technique was not adopted in any of the clinical studies [67].

Several validated HRQoL tools, both generic and disease-specific, are available for children suffering from JIA, each with some strengths and weaknesses [20,68,69,70]. In almost all the included studies, information on HRQoL was collected from the parents/caregivers, irrespective of the age of the enrolled patients. Only the study by Brunner et al. specified that the questionnaires were completed by adolescent patients themselves or by parents for younger children [48]. Although both the parent proxy and child self-reported outcomes have their pros and cons, results of parent proxy HRQoL measures may differ considerably from self-reports [71]. Indeed, parents of children suffering from chronic diseases tend to evaluate their children’s HRQoL as much poorer than it would be rated from the child’s own perspective [72]. Considering that children from seven years of age are capable of understanding and reporting to PCROs [73], whenever a child can respond, the child’s self-report should always be deemed preferable [74,75].

Although the gold standard in JIA-specific HRQoL assessment is currently the JAMAR, providing both self- and parent proxy reports, it was not administered in any of the included studies [56]. The most common JIA-specific tools adopted were PtGA and CHAQ. The first was applied for HRQoL evaluation, while the second one was used to measure functional outcomes, mainly for the disability index component [68,69,70]. Moreover, individual perception of overall pain and/or nocturnal back pain was recorded on a 0–100 VAS scale in most of the studies reporting on HRQoL.

Although PCROs are valuable components of holistic medical care, HRQoL information is lacking in a non-negligible proportion of recently published phase III trials. This may be due to the limited awareness of clinicians and researchers, or due to their concern about the time required to implement HRQoL assessment and the difficulties associated with interpreting the child’s point of view. Moreover, the lack of standardized tools across the studies makes comparing PCROs challenging; this implies the loss of relevant information about the added benefit provided by novel therapies. There is a need for straightforward guidelines to help investigators in designing and incorporating HRQOL assessment into JIA clinical trials with methodological robustness as well as in exhaustively reporting the results.

Some limitations of this systematic review have to be acknowledged. Despite the searching strategy applied, some studies could have been missed. We limited our search to peer-reviewed publications and excluded any trials that did not include any explicit definition of the study phase. Published abstracts were also excluded because of their limited information; the same was true of results posted in clinical registries. Thus, it cannot be ruled out that the current analysis could have underestimated the publication of HRQoL results. In addition, the checklist applied to assess the HRQoL results, despite its previous use as a minimum standard criterion in reporting HRQoL outcomes, is very simple; nevertheless, it could provide a useful indication of study quality.

## 5. Conclusions and Future Considerations

Based on the present results, the use of HRQoL questionnaires is still below expectations in pediatric rheumatology. Thus, there is an urgent need to include assessment of children’s HRQoL among the study endpoints of phase III clinical trials. The systematic incorporation of HRQoL measures in addition to traditional clinical outcomes would make it possible to consider the child’s perspective when comparing the studied treatments, with the ultimate goal of selecting the most tailored therapy on the individual level. However, the methodology of collecting and reporting HRQoL data appears to be heterogeneous in terms of type of instruments, analysis, and presentation of the results. Thus, it should be improved and standardized before high-quality results can be obtained and before said results can exert a major influence on clinical decision-making. In the era of precision medicine, every member of the scientific community should cooperate to improve the quality of research in pediatric rheumatology. This may be achieved by encouraging the authors of clinical trial protocols and publications to include all the relevant outcomes to ensure that they achieve an exhaustive evaluation of the added value of new treatments in line with the current regulatory guidance. This will enhance the reliability and the professional standing of future phase III trials in JIA and, in turn, the quality of evidence that supports clinicians’ choices in the practical setting.

## Figures and Tables

**Table 1:** Characteristics of primary publications.

Characteristic	*n*. Publications	% of Publications
**Year of Primary Publication**		
2020–2023	6	27.3
2016–2019	6	27.3
2012–2015	10	45.4
**Journal Impact Factor**		
Low (<10)	8	36.4
Intermediate (10–20)	5	22.7
High (>20)	9	40.9
**Type of Sponsor**		
For-profit	18	81.8
No-profit	4	18.2
**Type of JIA**		
PoliJIA	2	9.1
Polyarticular course JIA	8	36.4
ERA/PsJIA	4	18.2
sJIA	8	36.3
**Study design**		
Randomized withdrawal trial	12	54.6
Open-label long-term extension	5	22.7
Open-label single arm trial	5	22.7
**Type of Treatment**		
Janus Kinase inhibitor	2	9.1
TNF inhibitor	6	27.3
IL inhibitor	11	50.0
B/T cells inhibitor	3	13.6
**Study results**		
Positive	21	95.5
Negative	1	4.5

**Table 2:** Characteristics of phase III trials conducted in patients with JIA.

Authors	Year	Source Funding	Countries Involved	Type JIA	Age	Study Design	Masking	Experimental vs. Control Arm	Primary Endpoint	Results/Note	HRQoL as Endpoint
Brunner Ann Rheum Dis. [25]	2023	Sponsor	NA	Active ERA and PsJIA, inadequate response to csDMARDs or NSAIDs	≥2 to <18 years	RCT, superiority	Blinded	Subcutaneous secukinumab (75/150 mg in patients <50/≥50 kg) or placebo up to 100 weeks (85 pts vs. 86 pts) after 12-week open-label period of secukinumab	Time to JIA flare (worsening of 30% or more in at least 3 of 6 JIA/ACR core set variables, with one or none of the variables improving by 30%)	Positive	No
Ramanan Lancet. [26]	2023	Sponsor	75 centers of PRINTO in 20 countries	pc-JIA after failure of csDMARDs or bDMARDs	≥2 to <18 years	RCT, superiority	Blinded	Oral baricitinib vs placebo (82 pts vs. 81 pts) for 32 weeks after 12-week open-label, lead-in phase of baricitinib	Time to JIA flare	Positive	Yes
Ruperto Lancet. [27]	2021	Sponsor	64 centers of PRINTO and PRCSG in 14 countries	pc-JIA	≥2 to <18 years	RCT, superiority	Blinded	Oral tofacitinib vs. placebo (88 pts vs. 85 pts) for 26 weeks after 18-week open label, run-in phase with tofacitinib	JIA flare rate	Positive	Yes
Ruperto Rheumatol. [28]	2021	Sponsor	33 centers of PRINTO in 9 countries	pc-JIA	≥2 to <18 years	Single-arm	Open-label	I.V. golimumab at weeks 0, 4, then every 8 weeks through week 52 plus MTX weekly through week 28	Pharmacokinetics (golimumab concentrations over 8-week interval)	Positive	Yes
Nishimura Modern Rheumatol. [29]	2021	Sponsor	Japan	sJIA	≥2 to <20 years	Single-arm	Open-label	Canakinumab (4 mg/kg) every 4 weeks for 48 weeks	Proportion of patients achieving ACI Pedi 30 at 8 weeks	Positive	Yes
Quartier Arthritis Rheumatol. [30]	2021	Sponsor	45 centers of PRINTO and PRCSG in 16 countries	sJIA in clinical remission	≥5 to <16 years	RCT, not powered to compare the 2 arms	Open label	Subcutaneous canakinumab at a reduced dose (38 pts) versus subcutaneous canakinumab 4 mg/kg dose at prolonged intervals (37 pts)	At least 40% of patients remained in clinical remission for 24 consecutive weeks	No differences between the 2 tapering strategies	No
Hara Ped Rheumatol. [31]	2019	Sponsor	13 centers in Japan	pc-JIA with inadequate response to MTX or bDMARD	≥4 to <18 years	Single-arm	Open label	I.V. abatacept with or without MTX for 16 weeks (22 pts), followed by an ongoing long-term period	JIA-ACR 30 response rate	Positive	Yes
Foeldvari Arthritis Res Ther. [32]	2019	Sponsor	38 centers in 19 member countries of PRINTO	eoJIA, ERA, and PsA	≥2 to ≤17 years	6-year LTE of phase III trial (CLIPPER)	Open-label	Etanercept (once-weekly 0.8 mg/kg)	aJIA-ACR 50/70/90, based on the JIA core set variables	Positive	Yes
Opoka-Winiarska Clin Rheumatol. [33]	2018	No profit	Member Centers of PRINTO in Poland and Russia	pc-JIA with inadequate response to MTX	≥2 to <18 years	6-year LTE of phase III trial (CHERISH)	Open label	I.V. tocilizumab (8 mg/kg), intravenous infusion every 4 weeks	Safety (number and percentage of AEs, SAEs, and study drug-related AEs)	Positive Safety profile consistent with the pre- b CHERISH clinical trial.	No
Brunner Ann Rheum Dis. [34]	2018	Sponsor	33 centers of PRINTO and PRCSG in 12 world countries	pc-JIA	≥2 to <18 years	RCT, superiority	Blinded	Subcutaneous golimumab (30 mg/m^2^ body surface area every 4 weeks) or placebo (78 pts vs. 76 pts) for 32 weeks after an open label lead-in period of 16 weeks	JIA flare rate	Primary endpoint not met	No
Brunner Arthritis Rheumatol. [35]	2018	Sponsor	48 centers of PRINTO and PRCSG worldwide	pc-JIA	≥6 to <18 years	Single-arm	Open-label	Weekly subcutaneous abatacept treatment over 24 months	Abatacept steady-state serum trough concentration at 4 months	Positive	Secondary publication [36]
Ruperto Ann Rheum Dis [37]	2018	Sponsor	63 centers of PRINTO and PRCSG worldwide	Active sJIA	≥2 to ≤19 years	5-year LTE of 2 phase III trials [38]	Open-label	Canakinumab 4 mg/kg subcutaneously every 4 weeks (144 pts)	JIA-ACR 50/70/90, based on the JIA core set variables	Positive	No
Horneff Arthritis Rheumatol. [39]	2015	Sponsor	8 centers in Germany	Active ERA	≥6 to <18 years	RCT, superiority	Blinded	Etanercept (20 pts) vs. placebo (18 pts) for 24 weeks after a 24-week lead-in period with open label etanercept	JIA flare rate	Positive	Yes
Lovell Arthritis Rheumatol. [40]	2015	Sponsor	43 centers of PRINTO in Europe and America biologic agent that selectively modulates T cell stimulation.	Active JIA, inadequate response to ≥DMARD	≥6 to ≤17 years	7-year LTE of phase III trial	Open-label	I.V. abatacept (10 mg/kg) + MTX + corticosteroids (153 pts)	Safety	Positive	Yes
Burgos-Vargas Arthritis Care Res. [41]	2015	Sponsor	16 centers in Europe and Canada	ERA	≥6 to <18 years	RCT	Blinded	Adalimumab (31 pts) or placebo (15 pts) for 12 weeks	Change from baseline in percentage of active joints	Positive	Yes
Zhong Tohoku J Exp Med. [42]	2015	NA	Single-center, China	oligoJIA, sJIA, poliJIA	<16 years	RCT, superiority	Blinded	Etanercept (67 pts) vs. etanercept + blueberry juice placebo (67 pts) vs etanercept + placebo (67 pts) for 6 months	ACR20, ACR50, ACR70	Positive	Yes
Brunner Ann Rheum Dis. [43]	2015	No profit	58 centers of PRINTO and PRCSG in Europe, USA, Canada, Australia, Latin America, and Russia	pc-JIA with inadequate response to MTX	≥2 to <18 years	RCT, superiority	Blinded	Monthly I.V. tocilizumab (10 mg/kg or 8 mg/Kg mg in patients <30/≥30 kg) or placebo (82 vs. 81) up to 24 weeks after 16-week lead-in period of open-label tocilizumab	JIA flare rate	Positive	No
Ilowite Arthritis Rheumatol. [44]	2014	No profit	20 centers in USA	sJIA	≥18 months to ≤19 years	RCT, superiority	Blinded	Subcutaneous rilonacept for 24 weeks (36 pts) versus 4 weeks of placebo followed by 20 weeks of rilonacept (35 pts)	Time to response during the first 12 weeks (ACR Pedi 30, absence of fever, and corticosteroid taper)	Positive	Yes
Yokota J Rheumatol. [45]	2014	Sponsor	8 centers in Japan	Active sJIA	≥2 to <19 years	LTE of a phase III study, single-arm	Open label	Oral tocilizumab (50 pts) up to 5 years of follow-up	Safety	Positive Acceptable overall benefit/risk profile of tocilizumab given the severity of the disease and the corticosteroid tapering effects	No
Kingsbury Clin Rheumatol. [46]	2014	Sponsor	14 centers in EU and US	PoliJIA	≥2 to ≤4 years	Case series	Open-label	Adalimumab (24 mg/m^2^) every other week up to 120 weeks, with or without concomitant MTX (32 pts)	Safety	First study evaluating efficacy and safety of adalimumab in 2- to 4-year-old patients	Yes
De Benedetti NEJM. [47]	2012	Sponsor	43 centers of PRINTO and PRCSG worldwide	Active sJIA	≥2 to <18 years	RCT, superiority	Blinded	I.V. tocilizumab (12 mg/kg if the weight was <30 kg or 8 mg/kg if the weight ≥30 kg) or placebo every 2 weeks for 12 weeks	JIA-ACR 30 response rate	Positive	Secondary publication [48]
Ruperto NEJM. [38]	2012	Sponsor	63 centers of PRINTO and PRCSG worldwide	Active sJIA	≥2 to ≤19 years	trial 1: RCT, superiority trial 2: RCT, superiority	trial 1: blinded trial 2: blinded	trial 1: single subcutaneous dose of canakinumab (4 mg/Kg) or placebo trial 2: canakinumab every 4 weeks or placebo after open-label treatment with canakinumab for 12 to 32 weeks	trial 1: JIA-ACR 30 response rate trial 2: time to flare	trial 1: positive trial 2: positive	trial 1: no HRQoL trial 2: no HRQoL

ACR20, ACR30, ACR50 or ACR70 to reflect the 20%, 30%, 50%, or 70% improving levels in core set parameters of rheumatoid arthritis; AE: adverse event; SAE: serious adverse event; DMARD: disease-modifying antirheumatic drug; eoJIA: extended oligoarticular JIA; ERA: enthesitis-related arthritis; JIA: juvenile idiopathic arthritis; JIA-ACR: juvenile idiopathic arthritis-American College of Rheumatology; LTE: long-term extension phase; NSAID: nonsteroidal anti-inflammatory drug; MTX: methotrexate; pc-JIA: polyarticular course of JIA; PsJIA: psoriatic arthritis; PoliJIA: polyarticular JIA; HRQOL: health-related quality of life; RCT: randomized clinical trial; sJIA: systemic JIA. PRCSG: Pediatric Rheumatology Collaborative Study Group; PRINTO: Pediatric Rheumatology International Trials Organization; Pt: patient.

**Table 3:** HRQoL among the endpoints according to the study characteristics.

Characteristic	No. of Publications	HRQoL Included Among Endpoints, *n* (%)	HRQoL Not Included Among Endpoints, *n*. (%)
**Year of Primary Publication**			
2020–2023	6	4 (66.7)	2 (33.3)
2016–2019	6	2 (33.3)	4 (66.7)
2012–2015	10	6 (60.0)	4 (40.0)
**Journal Impact Factor**			
Low (<10)	8	5 (62.5)	3 (37.5)
Intermediate (10–20)	5	3 (60.0)	2 (40.0)
High (>20)	9	4 (44.4)	5 (55.6)
**Type of Sponsor**			
For-profit	18	10 (55.5)	8 (44.4)
No-profit	4	2 (50.0)	2 (50.0)
**Type of JIA**			
poliJIA	2	2 (100)	0 (0.0)
polyarticular course JIA	8	4 (50.0)	4 (50.0)
ERA/PsJIA	4	3 (75.0)	1 (25.0)
sJIA	8	3 (37.5)	5 (62.5)
**Study design**			
Randomized withdrawal trial	12	6 (50.0)	6 (50.0)
Open-label long-term extension	5	4 (80.0)	1 (20.0)
Open-label single-arm trial	5	2 (40.0)	3 (60.0)
**Type of Treatment**			
Janus Kinase inhibitor	2	2 (100)	0 (0.0)
TNF inhibitor	6	5 (83.3)	1 (16.7)
IL inhibitor	11	3 (27.3)	8 (72.7)
B/T cells inhibitor	3	2 (66.7)	1 (33.3)
**Study results**			
Positive	21	12 (57.1)	9 (42.9)
Negative	1	0 (0.0)	1 (100)

**Table 4:** Details of the methodology of HRQoL assessment and presentation of the results.

Characteristic	Number of Publications (%)
**HRQoL questionnaire (not mutually exclusive)**	
CHAQ/CHAQ-DI	8 (61.5)
PtGA	3 (23.1)
CHQ	2 (15.4)
Others	5 (38.5%)
**Pain perception**	
CHAQ questionnaire	1 (7.7)
VAS scale	7 (53.8)
**Modality of HRQoL analysis (not mutually exclusive)**	
Mean changes/scores	9 (69.2)
Median changes/scores	3 (23.1)
Others	3 (23.1)
**Missing data**	
Not imputed missing data	1 (7.7)
Not specified	8 (61.5)
Specified	4 (30.8)
**MCID**	
Specified	5 (38.5)
Not specified	8 (61.5)

CHAQ: Childhood Health Assessment Questionnaire; CHAQ-DI: Childhood Health Assessment Questionnaire Disability Index; CHQ: Child Health Questionnaire; MCID: minimal clinically important difference; PtGA: parent/patient global assessment; VAS: Visual Analogue Scale.

**Table 5:** Quality of HRQoL outcomes assessment and reporting according to Efficace et al. [23].

HRQoL Issue	Number of Publications with Criteria (%)
**Conceptual**	
A priori hypothesis stated	2 (15.4)
Rationale for instrument reported	0 (0.0)
**Measurement**	
Psychometric properties reported	3 (23.1)
Cultural validity verified	7 (53.8)
Adequacy of domains covered	10 (76.7)
**Methodology**	
Instrument administration reported	4 (30.8)
Baseline compliance reported	13 (100)
Time of assessments documented	10 (76.7)
Missing data documented	1 (7.7)
**Interpretation**	
Clinical significance	6 (46.2)
Presentation of results in general	12 (92.3)

## Data Availability

All data used in this study are available in the manuscript or its Supporting Material.

## Supplementary Materials

The following supporting information can be downloaded at: https://www.mdpi.com/article/10.3390/jcm14010254/s1, Table S1: Search strategy; Table S2: Health-related quality of life in the primary and secondary publications of trials with QoL as endpoint; Table S3: Methodological quality of HRQoL assessment.
