# Supplementary material for: Health-Related Quality of Life in Juvenile Idiopathic Arthritis: A Systematic Review of Phase III Clinical Trials
Source: J Clin Med. 2025 Jan 3;14(1):254. doi: 10.3390/jcm14010254 (PMC11721659; doi:10.3390/jcm14010254)
Supplement: Supplementary file 1 [file jcm-14-00254-s001.zip › jcm-3369519-supplementary.pdf]

Supplementary Materials:

Table S1. Search strategy.

"juvenile idiopathic arthritis"[All Fields] AND ("infant"[MeSH Terms] OR "infant"[All Fields] OR "child"[MeSH Terms] OR "child"[All Fields] OR "adolescent"[MeSH Terms] OR "[illegible] trial"[Publication Type] OR "randomized controlled trials as topic"[MeSH Terms] OR "randomized controlled trial"[All Fields] OR "controlled clinical trial"[All Fields] OR "controlled [illegible] trials as topic"[MeSH Terms] OR "clinical trial"[Publication Type] OR "clinical trial"[All Fields]).

Table S2. Health-related quality of life in the primary and secondary publications of trials with HRQoL as endpoint.

Authors	Primary/ secondary analysis	HRQoL as endpoint	HRQoL tools	Type of HRQoL analysis	HRQoL results in publication	MCID	Missing data	Drug approval	Available study protocol
Ruperto et al. (2023) [36]	Secondary analysis of Brunner et al. 2018 [35]	Primary	CHAQ-DI<br>PtGA<br>Pain<br>ALQ	Median change with respect to baseline<br><br>Proportions of patients with minimal clinically important improvement and absence of disability	Reported	Specified	Not imputed missing values	Early and sustained clinically relevant PRO improvements of SC abatacept in pJIA, also in very young children (i.e., those ages 2 to 5 years)	Yes
Ramanan et al. (2023) [26]	Primary	Secondary	CHQ-P50<br>CHAQ pain severity scale (0-100 VAS)	Mean change from baseline	Reported	for CHQ-PF50	Not specified	Yes (used only in adults with RA)	Yes

Brunner et al. (2021) [48]	Secondary analysis of Brunner et al. 2015 [43] and De Benedetti et al. 2012 [47]	Primary	CHAQ<br>CHQ-P50<br>CHQ-P50-PhS<br>CHQ-P50-PsS<br>Pain and well- being (100-mm VAS)	Mean value and SD<br>Frequencies of disability and pain categories over time	Reported	Specified	Not specified	I.V. tocilizumab significantly diminished patient disability and pain and improved of HRQoL for sJIA as well polyJIA	Yes
Ruperto et al. (2021) [27]	Primary	Secondary	CHAQ-DI	Mean value	Reported	Not specified	Not specified	Approval for oral administration	Yes
Nishimura et al. 2021 [29]	Primary	Secondary	CHAQ-DI<br>PtGA of overall well-being (0–100 mm VAS)	Mean value and SD	Reported	Not specified	Specified	New treatment option in Japan	No
Hara et al. (2019) [31]	Primary	Secondary	CHAQ-DI	Median improvement with respect to baseline	Reported	Not specified	Specified	New treatment option in Japan	No
Foeldvari et al (2019) [32]	Primary	Secondary	CHAQ<br>PtGA<br>Overall and nocturnal back pain 0-100-mm VAS) for ERA	Mean value and CI	Reported	Not specified	Specified	Open label treatment with ETN up to 6 years safe, well tolerated, and effective in patients with eoJIA, ERA, and PsA	No

Burgos-Vargas et al. (2015) [41]	Primary	Secondary	Patient's assessment of total back pain (0–100 mm VAS). Parent's assessment of patient's pain	Mean value and SD	Reported	Not specified	Not specified	Adalimumab may be an appropriate treatment option for patients with active ERA who have failed conventional treatment	No
Zhong et al. (2015) [42]	Primary	Secondary	Pain scale HAQ	Mean rank	Reported	Not specified	Not specified	Possible combination to reduce the side effects of etanercept	No
Lovell et al. (2015) [40]	Primary	Secondary	CHQ CHAQ-DI Children s Sleep Habits Questionnaire (CSHQ)	Mean value and SD	Reported	Specified	Specified	Long-term treatment with abatacept is associated with consistent safety relative to the short-term period and, in patients continuing treatment.	No
Horneff et al. (2015) [39]	Primary	Secondary	Patient's assessments of total and nocturnal back pain Parent s assessment of the patient s pain Parent s assessment of patient s overall well-being	Mean value and SD	Not reported	Specified	Not specified	Despite the fewer rates in children who continued treatment with respect to placebo, Treatment suspension may be a consideration for patients who achieve remission	No

Ilowite et al. (2014) [44]	Primary	Secondary	CHAQ-DI	Median and IQR	Reported (tables)	Not specified	Not specified	Rilonacept treatment facilitated corticosteroid tapering, patients who receive active drug earlier will respond sooner, on average, than patients who receive active drug later	No
Kingsbury et al. (2014) [46]	Primary	Secondary	Parent s Global Assessment of Pain (0–100 mm VAS)	Mean value and SD	Reported	Not specified	Not specified	First study evaluating efficacy and safety of adalimumab in 2 to 4 years old patients	No

ALQ: Activity Limitation Questionnaire; CHAQ: Childhood Health Assessment Questionnaire; CHAQ-DI: Childhood Health Assessment Questionnaire Disability Index; CHQ: Child Health Questionnaire.

Table S3. Methodological quality of HRQoL assessment.

	Domains										
	Conceptual		Methodology			Measurement			Interpretation		
**Authors**	**A priori hpothesis**	**Rationale for instrument reported**	**Psychometric properties reported**	**Cultural validity verified**	**Adequacy of domains covered**	**Instrument administration reported**	**Baseline compliance**	**Timing of assessment**	**Missing data**	**Clinical significance**	**Presentation of results in the text**	**Final score**
Ruperto et al. (2023) [36]	No	No	Yes	Yes	Yes	Yes	Yes	Yes	No	Yes	Yes	8
Ramanan et al. (2023) [26]	No	No	No	Yes	Yes	Yes	Yes	Yes	No	Yes	Yes	7
Brunner et al. (2021) [48]	Yes	No	Yes	Yes	Yes	Yes	Yes	Yes	No	Yes	Yes	9

Ruperto et al. (2021) [27]	No	No	No	Yes	Yes	No	Yes	Yes	No	No	Yes	5
Nishimura et al. 2021 [29]	No	No	No	Yes	Yes	No	Yes	Yes	No	No	Yes	5
Hara et al. (2019) [31]	No	No	No	No	Yes	No	Yes	Yes	No	Yes	Yes	5
Foeldvari et al (2019) [32]	No	No	No	Yes	Yes	No	Yes	Yes	No	No	Yes	5
Burgos-Vargas et al. (2015) [41]	No	No	No	No	Yes	No	Yes	Yes	No	No	Yes	4
Zhong et al. (2015) [42]	No	No	No	No	Yes	No	Yes	Yes	No	Yes	Yes	5
Lovell et al. (2015) [40]	Yes	No	Yes	Yes	Yes	Yes	Yes	Yes	Yes	Yes	Yes	10
Horneff et al. (2015) [39]	No	No	No	No	No	No	Yes	No	No	No	No	1
Ilowite et al. (2014) [44]	No	No	No	No	No	No	Yes	No	No	No	No	1
Kingsbury et al. (2014) [46]	No	No	No	No	No	No	Yes	No	No	No	Yes	2
